# Receive diversity based transmission data rate optimization for improved network lifetime and delay efficiency of Wireless Body Area Networks

**DOI:** 10.1371/journal.pone.0206027

**Published:** 2018-10-25

**Authors:** K. Senthil Kumar, R. Amutha, M. Palanivelan, D. Gururaj, S. Richard Jebasingh, M. Anitha Mary, S. Anitha, V. Savitha, R. Priyanka, Amruth Balachandran, H. Adithya, Asher Shaji, Anchana C.

**Affiliations:** 1 Department of Electronics and Communication Engineering, Rajalakshmi Engineering College, Chennai, India; 2 Department of Electronics and Communication Engineering, SSN College of Engineering, Chennai, India; Dalian University of Technology, CHINA

## Abstract

Wireless Body Area Network (WBAN) has become the emerging technology due to its ability to provide intelligent and cost-effective healthcare monitoring solution. The biological sensors used in WBAN are energy-constrained and required to be functional for a longer duration. Also, the sensed data should be communicated in reasonable time. Therefore, network lifetime and delay have become the primary concerns in the design of WBAN. In this paper, Receive Diversity based Transmission Data Rate Optimization (RDTDRO) scheme is proposed to improve the network lifetime and delay efficiency of Multi level-Quadrature Amplitude Modulation (M-QAM) based WBAN. In the proposed RDTDRO scheme, minimum energy consumption is ensured by optimizing the transmission data rate with respect to a given transmission distance and number of receive antennas while satisfying the Bit Error Rate (BER) requirements. The performance of proposed RDTDRO is analyzed in terms of network lifetime and delay difference and is compared with conventional Baseline and Rate optimized schemes. The results show that at a transmission distance of 0.3 m, the proposed RDTDRO scheme with a receive diversity order of 4 achieves 1.30 times and 1.27 times improvement in network lifetime over conventional Baseline and Rate optimized schemes respectively. From the results, it is also evident that at a transmission distance of 0.3 m, the proposed RDTDRO scheme with a receive diversity order of 4 is delay efficient as it achieves delay difference of 0.75 *μ*s and 0.29 *μ*s over conventional Baseline and Rate optimized schemes respectively.

## Introduction

Wireless Body Area Network (WBAN) is a sensor network which is intended to connect various medical sensors and devices, placed in and out of a human body. In recent studies, WBAN has been supported widely due to their extraordinary potential in improving the quality of life and human health [1], [2], [3], [4], [5], [6]. For instance, diagnosis of diseases, medical treatment, fine-tuning sports persons’ schedule, and public safety are some of the applications. The sensors deployed in WBAN detects important biological signals like body temperature, blood pressure and Electrocardiogram (ECG) etc., and transmit the data to Body Control Unit (BCU) or remote medical server for analysis and diagnosis.

The WBAN is expected to offer long-standing health monitoring without affecting their routine work. In WBAN, the sensor nodes are powered by batteries for their functions like sensing the important biological signals, data processing and communicating the data [2]. The total energy consumption of a sensor node comprises two components-transmission energy and circuit energy [7], [8]. The transmission energy is the energy consumed during the transmission of data during communication. The circuit energy is the energy consumed by the circuit blocks during their operation. The transmission energy consumption depends mainly on the parameters like transmission distance, transmission data rate, reliability and path loss. The circuit energy consumption mainly depends on the transmission data rate and the hardware [7], [8]. To avoid inconvenience to the person, the batteries are made small as the sensors are placed on or implanted inside the human body. The size of the batteries limits the battery capacity. Therefore, the energy consumed by the sensor node needs to be minimized. In general, the sensors of WBAN are able to communicate up to 2 m [1] (i.e. transmission distance). Though the advent of low power Very Large Scale Integration (VLSI) technology made circuit power consumption increasingly smaller, it is comparable to the transmission energy due to smaller transmission distance involved with WBAN [9]. Therefore, the circuit energy consumption also should be taken into consideration during transmission data rate optimization.

Network lifetime is defined as the time at which the first node in the network runs out of energy to send a packet because to lose a node could mean that the network could lose some functionality [10]. Delay is the amount of time it takes for the head of the signal to travel from the sender to the receiver. It can be computed as the ratio between the link length and the propagation speed in the specific medium [11]. It is the amount of time that a WBAN would be fully operative. The sensor nodes are powered by small batteries and hence they are energy limited. Therefore, delay efficiency and improving network lifetime becomes vital in WBAN.

In the recent past, a significant amount of research has been carried out in the improvement of delay efficiency and network lifetime in WBAN. In [11], a delay efficient algorithm for the minimization of delay using data aggregation is presented. The delay profile of a wireless sensor network is determined in [12]. In [13], an optimal coordinator deployment method is proposed for lifetime maximization in wireless body sensor networks. A method for the organization of energy efficient wireless sensor network is presented in [14] to improve the network lifetime. Energy efficient approach which optimizes the transmission data rate according to the transmission distance for M-PSK based WBAN is proposed in [15]. In [16], the authors demonstrated the enhancement in network lifetime of a WBAN by adopting Bayesian game formulation. They also manifested the reduction in delay by employing ant colony optimization approach.

Diversity is a technique which is used to overcome the adverse effect of multipath fading [17]. Receive diversity uses multiple receive antennas for signal reception. A transmission strategy to minimize delay while taking the effect of circuit energy consumption into account and utilizing diversity is proposed in [18] and [19]. The improvement in energy efficiency that can be achieved by utilizing diversity in conjunction with space-time coding on wireless sensor network is demonstrated in [20]. In [21] and [22], the energy efficiency that can be achieved by utilizing diversity in conjunction with channel coding on wireless sensor network is illustrated. An algorithm which optimizes the transmission distance for improving the energy efficiency of a wireless sensor network by utilizing diversity is presented in [23]. The energy efficiency of MPSK based WBAN is investigated in [24]. Therefore, there is a scope for improvement in delay efficiency and network lifetime of a WBAN by utilizing diversity and proper selection of transmission distance and transmission data rate.

The main contributions of the paper are

1. Receive Diversity based Transmission Data Rate Optimization (RDTDRO) scheme is proposed to improve the network lifetime and delay efficiency of M-QAM based WBAN.
2. Demonstrated the superior performance of proposed RDTDRO scheme in terms of network lifetime and delay efficiency over conventional schemes.

The rest of the paper is organized as follows. An energy model for M-QAM based WBAN is presented in section 2. Section 3 describes the delay model for M-QAM based WBAN. Section 4 presents the simulation results. Finally, section 5 concludes the paper.

## M-QAM basedEnergy model

The total energy consumed per bit by a sensor node [25] is defined as

$$E_{bit\_total}=\frac{P_{active}T_{active}+P_{sleep}T_{sleep}}{L}$$ (1)

where *P*_*active*_ is the power required by a sensor node when is in the active state for a period of duration of *T*_*active*_, *P*_*sleep*_ is the power required by a sensor node when is in the sleep state for a period of duration of *T*_*sleep*_ and *L* is the number of transmitted bits by the sensor node.

The allowable time limit *T*, within which *L* bits are to be transmitted, is defined as

$$T_{active}+T_{sleep}=T$$ (2)

The power required by a sensor node when is in the active state comprises of two components—power required by the Power Amplifier *P*_*amplifier*_ and power required by all other circuit blocks along the signal path *P*_*circuit*_ [8] and is defined as

$$P_{active}=P_{amplifier}+P_{circuit}$$ (3)

The power required by the power amplifier [8] is defined as

$$P_{amplifier}=\left(\frac{\xi }{\eta }\right)P_{TX}$$ (4)

where *ξ* is the peak-to-average ratio, *η* is the drain efficiency of the power amplifier and *P*_*TX*_ is the transmission power.

**Fig 1:**
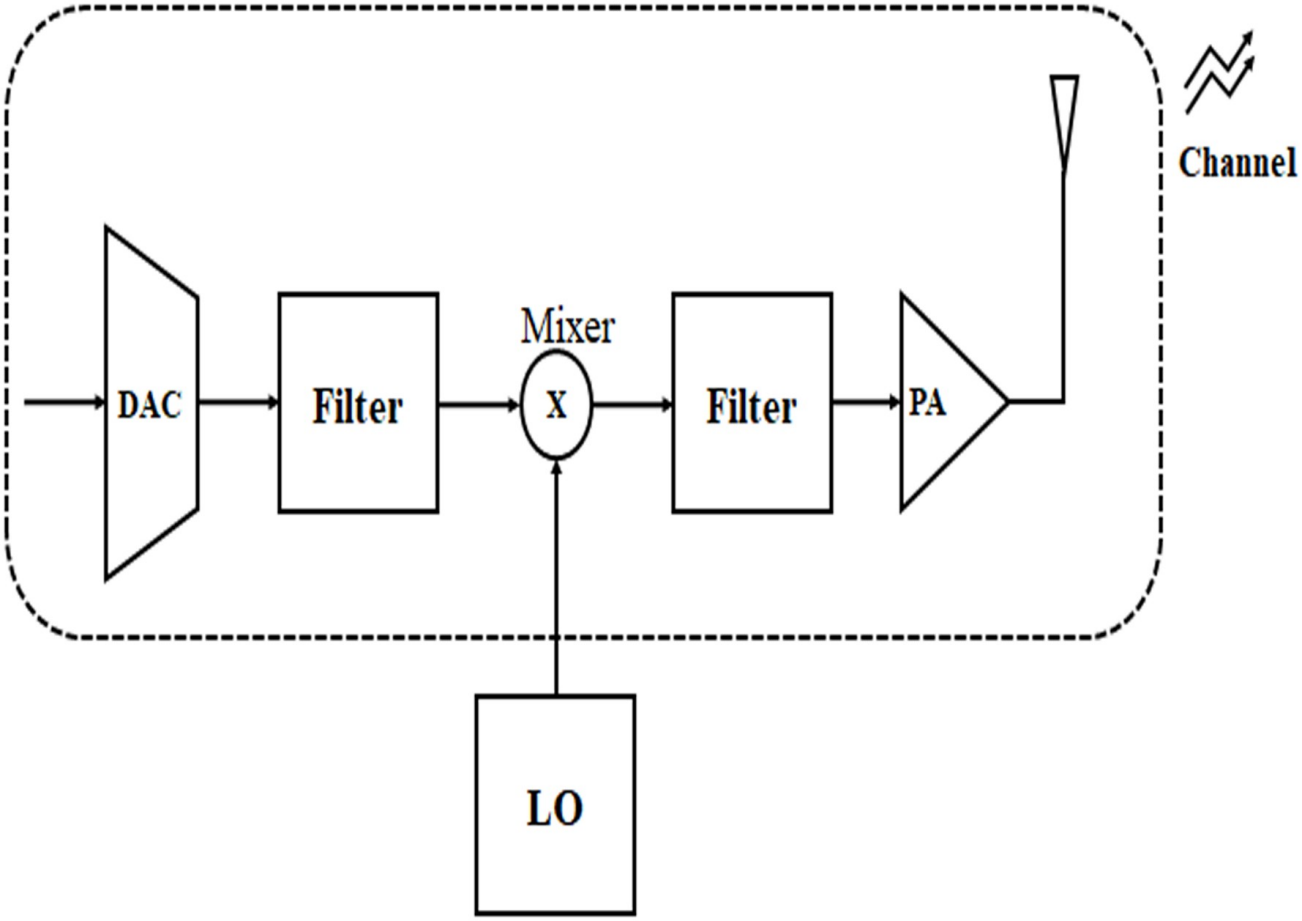
Analog circuit blocks of a sensor node transmitter.

The Fig 1 shows the anolog circuit blocks of a transmitter of a sensor node. In order to keep the system model simple, the baseband circuit blocks are not considered. Due to size constraints, the sensor node can accommodate only single antenna.

**Fig 2:**
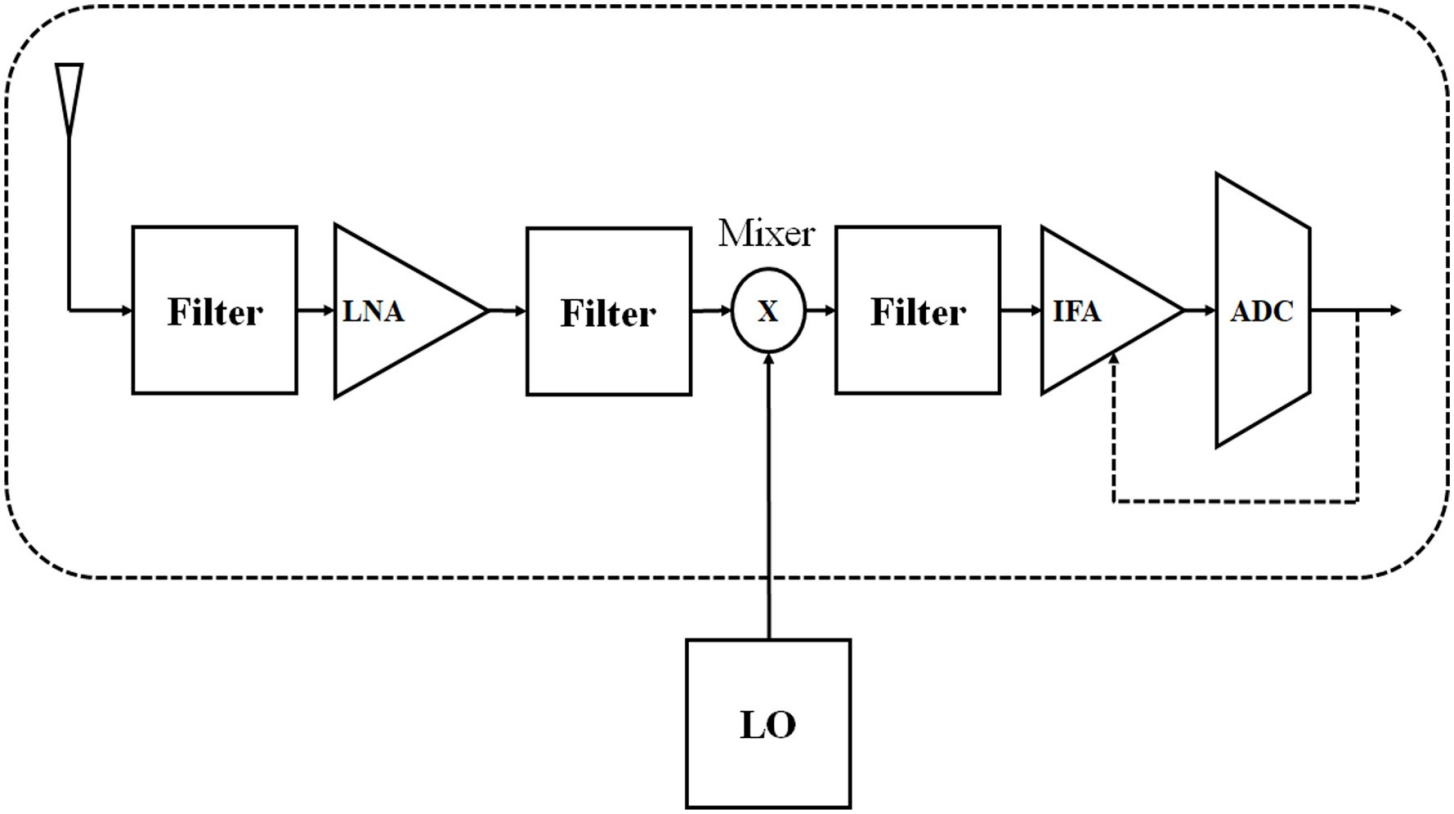
Analog circuit blocks of a BCU receiver.

The Fig 2 shows the anolog circuit blocks of a BCU at the receiver. Unlike the sensor node (i.e. as there is no size restriction), the BCU can support more than one antenna.

The transmission power *P*_*TX*_ of (4) is defined by link power budget relationship and the path loss model specified for WBAN by [9] as

PTX=E¯bit_RX_BER(R(4π)210((b_loss+N_loss+Ml+Nf)10)d(a_loss10)10(GTXGRX10)λ(a_loss10)) (5)

where ${\bar{E}}_{bit\_RX\_BER}$ is the energy required per bit at the receiver for a given Bit Error Rate (BER), *R* is the transmission data rate, *a*_*loss* and *b*_*loss* are coefficients of linear filtering, *N*_*loss* is a normally distributed variable with standard deviation *δ*_*N*_, *M*_*l*_ is the link margin, *N*_*f*_ is the receiver noise figure, *d* is the transmission distance, *G*_*TX*_ is the transmitter antenna gain, *G*_*RX*_ is the receiver antenna gain and *λ* is the carrier wavelength.

Assume that WBAN employs Multilevel-Quadrature Amplitude Modulation (M-QAM). Then, at the receiver, the bit error rate *P*_*BER*_ for M-QAM (M ≥ 4) [26] can be expressed as

pBER=4b(1−12b2)Q(3bγRX_BERM−1) (6)

where *b* is the number of bits used to represent a symbol, Q(.) is the Q-function, *M* is the modulation order and *γ*_*RX*_*BER*_ is the required Signal to Noise Ratio (SNR) at the receiver for a given BER.

A system with receive diversity [17] i.e. Single-Input-Multiple-Output (SIMO) has an SNR which is defined as

$$\gamma_{RX\_BER}=\frac{{\left\|H_{1\times M_{r}}\right\|}^{2}{\bar{E}}_{bit\_RX\_BER}}{N_{0}}$$ (7)

where *H* is a row vector of size 1×*M*_*r*_, ${\left\|H_{1\times M_{r}}\right\|}^{2}$ is the channel gain, *M*_*r*_ is the number of antennas at the receiver and *N*_0_ is the single sided thermal noise power spectral density. Since more transmitting nodes may cause inconvenience to the patient, the number of transmitting nodes is limited to one. As there may not be a size or energy constraint at the receiver, the receiver is assumed to have more than one antenna. Thus, a system with receive diversity is considered in this paper.

By substituting (7) in (6), the (6) can be written as

pBER=4b(1−12b2)Q(3b‖H1×Mr‖2E¯bit_RX_BER(M−1)N0) (8)

By applying Chernoff bound, the (8) can be modified as

pBER≤2b(1−12b2)exp(1.5b‖H1×Mr‖2E¯bit_RX_BER(M−1)N0) (9)

From (9) and by assuming equality, the ${\bar{E}}_{bit\_RX\_BER}$ can be expressed as

$${\bar{E}}_{bit\_RX\_BER}=\frac{(M-1)N_{0}}{1.5b{\left\|H_{1\times M_{r}}\right\|}^{2}}k(b)$$ (10)

where k(b)=ln(bpBER2(1−12b2)) is a function of *b*.

By substituting (10) in (5), the transmission power can be expressed as

PTX=Dξ(M−1)d(a_loss10)k(b)Rηb‖H1×Mr‖2 (11)

where D=(4π)210((b_loss+N_loss+Ml+Nf+N0)10)1.5(10(GTXGRX10)λ(a_loss10)) is a constant.

The required power of all circuit blocks along the signal path at the transmitter except power amplifier [8] of (3) is defined as

$$P_{circuit}=P_{DAC}+P_{mixer}+P_{filter}+P_{synthesizer}$$ (12)

where *P*_*Dac*_, *P*_*mixer*_, *P*_*filter*_ and *P*_*synthesizer*_ are the power consumption values for the Digital-to-Analog Converter (DAC), mixer, active filter at the transmitter and frequency synthesizer respectively.

The transmission data rate *R* [15] can be expressed as

$$R=\frac{L}{T}$$ (13)

By substituting (3), (4), (11), (12) and (13) in (1), the total energy consumed per bit can be expressed as

Ebit_total=Dξ(M−1)d(a_loss10)k(t)ηb‖H1×Mr‖2+Pcircuit−PsleepR+PsleepL (14)

The bandwidth for M-QAM [15] is given by

$$B=\frac{1+\alpha }{b}R$$ (15)

where *α* denotes the roll-off factor of the pulse shaping filter.

By substituting (15) in (14), the (14) can be written as

Ebit_total=Dξ(2(1+α)RB−1)Bd(a_loss10)v(R)η‖H1×Mr‖2(1+α)R+Pcircuit−PsleepR+PsleepL (16)

where v(R)=ln((1+α)RpBER2B(1−12((1+α)R2B))) is a function of *R*.

In (16), the first term corresponds to transmission energy and the rest corresponds to circuit energy consumption. From (16), it is clear that transmission energy is a function of transmission distance, channel gain and transmission data rate. It is also clear that the circuit energy consumption is a function of transmission data rate and independent of transmission distance and channel gain. Therefore, both the transmission distance and transmission data rate are to be optimized for a given receive diversity (the receive diversity which in turn determines the channel gain) to achieve minimum energy consumption.

### Maximum transmission distance

It is clear from (16) that the total energy per bit comprises of transmission energy and circuit energy. The circuit energy consumption becomes comparable to transmission energy and even dominates [27] since the transmission distance is short in WBAN. Therefore, it is clear that there exists a limit on maximum transmission distance *d*_max_ up to which the circuit energy consumption dominates the transmission energy. Also, it is clear that the circuit energy consumption decreases with increase in transmission data rate. Therefore, the total energy consumption can be reduced by optimizing the transmission data rate as long as the transmission distance *d* is lesser than maximum transmission distance (i.e. *d* ≤ *d*_max_) for a given diversity order. (Note: At the transmitter side, in the total energy calculation, the circuit energy consumption of the transmitter side circuit blocks only considered, as the lifetime of the sensor node is determined only by its circuit energy consumption and transmit energy consumption. Since BCU is considered to be energy unconstrained, the increase in circuit energy consumption at the BCU due to increase in diversity order is ignored).

From (16), it is clear that for a given transmission distance and diversity order, the total energy consumption is a function of transmission data rate and is strictly convex with the transmission data rate as long as *d* ≤ *d*_max_.

The problem to optimize the total energy consumption can be summarized as follows:

$$\text{Minimize}\;E_{bit\_total}$$

$$\begin{array}{ll} & R_{\text{min}}\leq R\leq R_{\text{max}}\\ \text{Subject}\;\text{to} & d\leq d_{\text{max}}\\ & 2\leq M_{r} \end{array}$$ (17)

where *R*_min_ and *R*_max_ are the minimum and maximum transmission data rate respectively. This is an optimization problem of variable *R* and the following proposition can be obtained.

*Proposition*:

The total energy consumption *E*_*bit*___*total*_ is dependent on the transmission data rate *R* and the whole function is convex and has a minimum value.

*Proof*: The value of the second derivative of (16) with respect to *R* results in a positive value and is expressed as

$$\frac{\partial^{2}E_{bit\_total}}{\partial R^{2}}>0$$ (18)

Hence, *E*_*bit*___*total*_ is convex and has a minimum value.

From (16), it can be understood that for a sensor node at the transmitter side, the transmission energy increases with increase in transmission data rate and the circuit energy consumption decreases increase in transmission data rate and hence strictly convex with *R* for a given diversity order. Therefore, the maximum transmission distance *d*_max_ can be found from the derivative of *E*_*bit*___*total*_ with respect to *R* at the minimum transmission data rate *R*_min_.

Using (13) and (15) and with *b* ≥ 2 for M-QAM, the minimum transmission data rate [15] can be expressed as

$$R_{\text{min}}=\text{max}\left\{\frac{L}{T},\frac{2B}{1+\alpha }\right\}$$ (19)

By combining (16) with (19), the maximum transmission distance is obtained as

$$d_{\text{max}}=\left\{d:\frac{\partial E_{bit\_total}}{\partial R}{|}_{R=R_{\text{min}}}=0\right\}$$ (20)

In order to minimize the total energy, the range of transmission data rate to make *R* ∈[*R*_min_, *R*_max_] with maximum transmission data rate *R*_max_ is to be determined.

In general, the WBAN devices have to transmit low power to protect the safety of human body and to reduce interference with other devices. The transmit power *P*_*TX*_ must satisfy *P*_*TX*_ ≤ *P*_max_ where *P*_max_ is the maximum transmit power set by the local regulatory bodies [28]. By using (5) and (10), the maximum transmission data rate is defined as

Rmax={R:D(2(1+α)RB−1)Bd(a_loss10)v(R)(1+α)R=Pmax} (21)

By assuming the maximum transmit power as 1.5 W [9] as specified by the Federal Communication Commission (FCC) together with the parameters listed in Table 1 [7], [9], [27], [28], the maximum transmission distance is computed as

$$d_{\text{max}}={\left[\frac{\left(P_{circuit}-P_{sleep}\right)\eta }{R^{2}\xi D}\frac{\frac{{\left(1+\alpha \right)}^{2}R^{2}}{B^{2}}}{\left[\frac{\left(1+\alpha \right)R}{B}\left(e(R)+f(R)\right)-g(R)\right]}\right]}^{\frac{10}{a\_loss}}$$ (22)

where $e(R)=\left(2^{\frac{\left(1+\alpha \right)R}{B}}-1\right)\left(\frac{1}{R}-\frac{1}{1-2^{\frac{-\left(1+\alpha \right)R}{2B}}}\frac{\left(1+\alpha \right)}{2B}2^{\frac{-\left(1+\alpha \right)R}{2B}}\text{log}\;2\right)$, $f(R)=\frac{\left(1+\alpha \right)Rv(R)2^{\frac{\left(1+\alpha \right)R}{B}}\text{log}\;2}{B}$ and $g(R)=-\left(2^{\frac{\left(1+\alpha \right)R}{B}}-1\right)v(R)$ are functions of *R*.

**Table 1:** Simulation parameters.

Parameter	Value	Parameter	Value
*ξ*	1	*α*	0.25
*G*_*TX*_*G*_*RX*_	5 dBi	*N*_0_/2	-171 dBm/Hz
*B*	400 kHz	*L*	2 kbits
c_loss	15.5	d_loss	5.38
*f*_*c*_	951.1 MHz	*λ*	0.3154 m
*P*_*BER*_	10^−6^	*η*	0.5
*P*_*circuit*_	12.5 mW	*P*_*sleep*_	0.5 mW
*M*_*l*_	40 dB	*N*_*f*_	10 dB
G_loss	5.35	*δ*_*n*_	5.35

### Network lifetime model

Let us assume that a sensor node transmits *L* bits of data, each time (i.e. round) it senses. Let *E*_*L*_(*i*) = *L* × *E*_*bit*_*total*_ be the total energy consumed for transmitting *L* bit of data during *i*^th^ round of transmission.

The residual energy of a sensor node is given by (23) as

$$residual\;energy(j)=initial\;energy-\sum_{i=1}^{j}E_{L}(i)$$ (23)

where *i* and *j* are integers.

The network lifetime is defined as the number of times (i.e. rounds) a node can transmit a set of data before its battery completely drains (i.e. zero residual energy) and is expressed as

Networklifetime={j:residualenergy(j)=0} (24)

### Delay model

The total delay suffered by an MPSK based on WBAN can be expressed as

$$\text{Delay}=\frac{1}{\text{B}}\left[\frac{\text{Number}\;\text{of}\;\text{bits}\;\text{to}\;\text{be}\;\text{transmitted}}{\text{constellation}\;\text{size}}\right]$$ (25)

$${\text{Delay}\;\text{Difference}=\text{Delay}}_{\text{conventional}}{-\text{Delay}}_{\text{proposed}}$$ (26)

where Delay_conventional_ is the delay suffered by the conventional scheme and Delay_proposed_ is the delay suffered by the proposed RDTDRO scheme. A positive value of delay difference indicates the proposed scheme is delay efficient. From (23), it can be understood that to minimize delay of the M-QAM based WBAN can be reduced by using higher constellation size i.e. higher transmission data rate. The optimum transmission data rate can be increased, for a given transmission distance, by increasing the diversity order.

## Results and discussion

In this work, Channel Model CM3 which covers frequency band 950–956 MHz is considered. The WBAN is simulated using MATLAB version 8 according to the specifications of IEEE 802.15.6 standard. For *d* ≤ *d*_max_, the total energy consumption can be minimized by optimizing *R* through an appropriate convex optimization algorithm, which is termed as conventional Rate Optimized scheme [15]. The total energy consumption can be minimized further by the proposed RDTDRO scheme. In addition, the scheme which uses fixed transmission data rate *R*_min_ termed as conventional Baseline scheme is also considered for fair comparison. To keep the experimental setup simple, single sensor node (having single antenna) is considered at the transmitter side. The BCU at the receiver side is assumed to have single antenna (i.e. *M*_*r*_ = 1) for conventional schemes (i.e. Baseline and Rate optimized schemes) and more than one antenna (i.e. *M*_*r*_ = 2, 3 & 4) for the proposed RDTDRO scheme. Only single hop transmission (i.e. between sensor and BCU) is considered in the simulation. Multihop transmission (i.e. through relay nodes) is not considered to keep the experimental setup simple. The initial energy of the battery is considered as 5 J as in [1].

**Fig 3:**
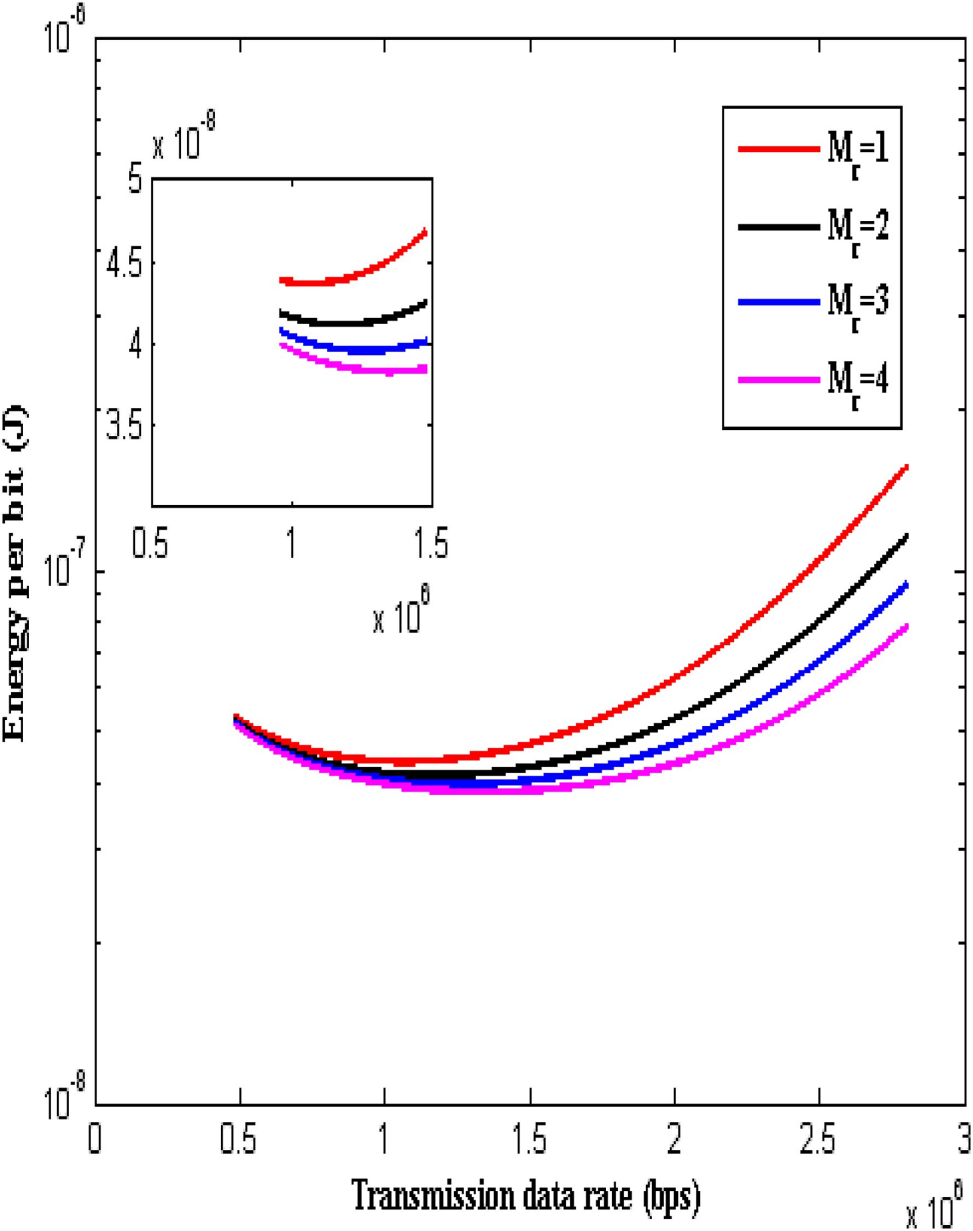
Energy consumption per bit over transmission data rate.

The total energy consumption per bit over transmission data rate for a transmission distance of *d* = 0.1 m with the number of receive antennas *M*_*r*_ = 1, 2, 3 and 4 is shown in Fig 3. In order to have the clear visualization of the influence of transmission data rate optimization and receive diversity on the total energy consumption, the transmission distance is considered as *d* = 0.1 m. From the Fig, it is observed that the total energy consumption per bit decreases initially with the increase in transmission data rate, reaches a minimum value and then increases for a given diversity value. The transmission data rate corresponding to the minimum total energy consumption is selected as the optimum transmission data rate *R*_*opt*_. For instance, the total energy consumption with *M*_*r*_ = 1 becomes minimum (i.e. *E*_*bit*_*total*_ = 4.46 × 10^−8^ J) at transmission data rate *R* = 1.03 × 10^6^ Hz. Therefore the optimum transmission data rate with *M*_*r*_ = 1 is *R*_*opt*_ = 1.03 × 10^6^ Hz. It is also observed that optimum transmission data rate increases and the value of minimum total energy consumption decreases with increase in diversity value. For instance, the total energy consumption is minimum (i.e. *E*_*bit*_*total*_ = 4.17 × 10^−8^ J) at transmission data rate *R* = 1.14 × 10^6^ Hz with *M*_*r*_ = 2. The total energy consumption is minimum (i.e. *E*_*bit*_*total*_ = 4.04 × 10^−8^ J) at transmission data rate *R* = 1.21 × 10^6^ Hz with *M*_*r*_ = 3. The total energy consumption is minimum (i.e. *E*_*bit*_*total*_ = 3.96 × 10^−8^ J) at transmission data rate *R* = 1.26 × 10^6^ Hz with *M*_*r*_ = 4.

**Fig 4:**
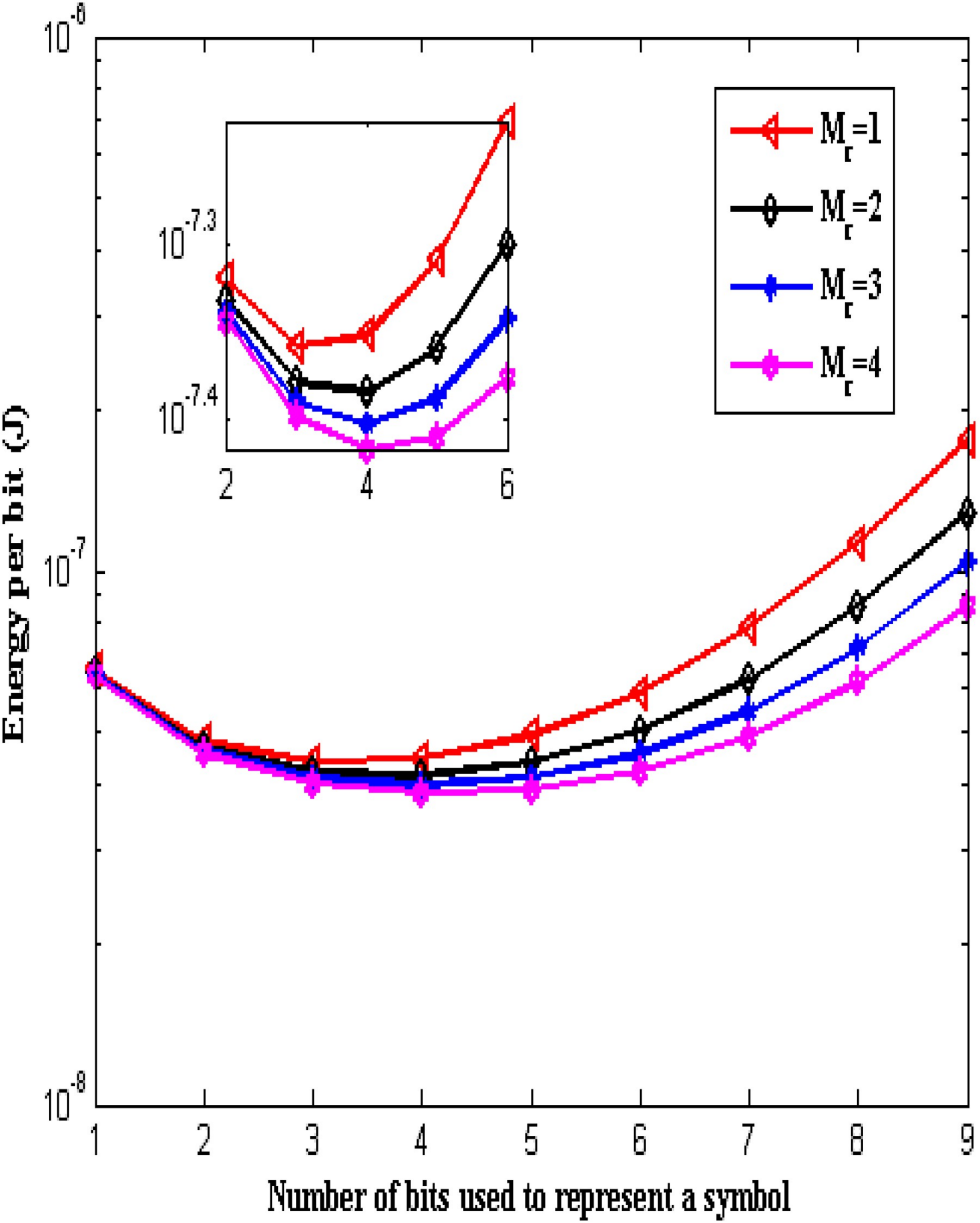
Energy consumption per bit over number of bits used to represent a symbol.

The total energy consumption per bit over the number of bits used to represent a symbol *b* for a transmission distance of *d* = 0.1 m with the number of receive antennas *M*_*r*_ = 1, 2, 3 and 4 is shown in Fig 4. From the Fig, it is observed that the total energy consumption per bit decreases initially with the increase in the number of bits used to represent a symbol, reaches a minimum value and then increases for a given diversity value. The number of bits used to represent a symbol corresponding to the minimum total energy consumption is selected as the optimum the number of bits to represent a symbol. From the Fig, it is observed that the total energy consumption becomes minimum (i.e. *E*_*bit*_*total*_ = 4.38 × 10^−8^ J) at *b* = 3 for *M*_*r*_ = 1. Thereforethe optimum number of bits for representing a symbol is *b* = 3 for *M*_*r*_ = 1. It is also observed that the optimum number of bits to represent a symbol increases and the value of minimum total energy consumption decreases with increase in diversity value. For instance, the minimum total energy consumption is 4.13 × 10^−8^ J at *b* = 4 with *M*_*r*_ = 2. The minimum total energy consumption is 3.95 × 10^−8^ J at *b* = 4 with *M*_*r*_ = 3. The minimum total energy consumption is 3.83 × 10^−8^ J at *b* = 4 with *M*_*r*_ = 4.

**Fig 5:**
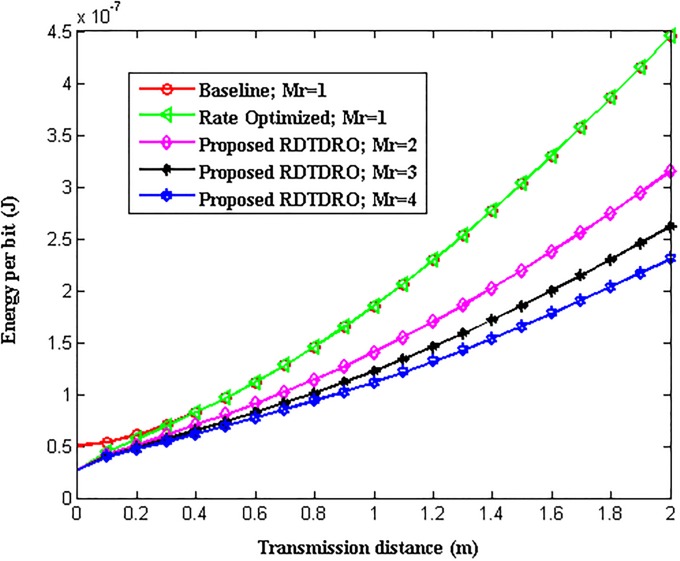
Energy consumption per bit over distance.

The total energy consumption per bit of M-QAM based WBAN using conventional Baseline, Rate optimized schemes with *M*_*r*_ = 1 and proposed RDTDRO schemes with *M*_*r*_ = 2, 3 and 4 over transmission distance is shown in Fig 5.

From the Fig, it can be observed that the total energy consumption of all the schemes increases with increase in transmission distance. The transmission data rate *R* for the conventional Baseline scheme is set equal to *R*_min_ which is 480 kHz and fixed irrespective of transmission distance. Therefore the total energy consumption of the conventional Baseline scheme is higher at all the transmission distance values considered when compared to other schemes. The conventional Rate optimized scheme uses optimum transmission data rate *R*_*opt*_ for every distance value. As the conventional Rate optimized scheme does not utilize the receive diversity (i.e. with *M*_*r*_ = 1 only), the optimum transmission data rate becomes 480 KHz above 0.5 m. Hence, the total energy consumption of conventional Rate optimized scheme is lesser than the total energy consumption of the conventional Baseline scheme up to 0.5 m. Above 0.5 m, the total energy consumption of both the conventional schemes become equal. The total energy consumption of the conventional schemes is higher at all the transmission distance values considered when compared to proposed RDTDRO scheme with *M*_*r*_ = 2, 3 and 4. The proposed RDTDRO scheme utilizes receive diversity in addition to transmission data rate optimization and hence their total energy consumption is lesser than conventional Baseline and Rate optimized scheme with *M*_*r*_ = 1. The transmission data rate optimization is beneficial for the proposed RDTDRO schemes with *M*_*r*_ = 2, 3 and 4 up to *d* = 0.7 m, *d* = 0.8 m and *d* = 0.8 m respectively. Above these distances, as the *R*_*opt*_ becomes equal to *Rmin*, the energy saving is only because of the receive diversity. From the Fig, it is clear that for a given transmission distance, the total energy consumption decreases with increase in the order of the receive diversity. From the Fig, it is also clear that the total energy consumption of proposed RDTDRO with *M*_*r*_ = 4 is lesser when compared to other schemes at any given distance.

Table 2 shows optimum transmission data rate for M-QAM based proposed RDTDRO and conventional schemes at various distances. From the table, it is clear that transmission data rate optimization is possible only for smaller distances. It is also observed that the optimum transmission data rate is higher for higher receive diversity.

**Table 2:** Optimum transmission data rate in kbps for M-QAM based schemes at various distances.

Transmission distance *d*	Optimum transmission data rate in kbps				
	Conventional		Proposed		
	Baseline (*M*_*r*_ = 1)	Rate optimized (*M*_*r*_ = 1)	RDTDRO (*M*_*r*_ = 2)	RDTDRO (*M*_*r*_ = 3)	RDTDRO (*M*_*r*_ = 4)
**0.1 m**	480.0	1031.9	1146.7	1213.4	1261.5
**0.2 m**	480.0	754.8	852.7	910.4	952.3
**0.3 m**	480.0	615.4	702.2	754.0	791.8
**0.4 m**	480.0	527.3	606.0	653.3	688.0
**0.5 m**	480.0	480.0	537.6	581.4	613.6
**0.6 m**	480.0	480.0	485.0	526.7	556.9
**0.7 m**	480.0	480.0	480.0	483.3	511.7
**from 0.8 m to 2 m**	480.0	480.0	480.0	480.0	480.0

**Fig 6:**
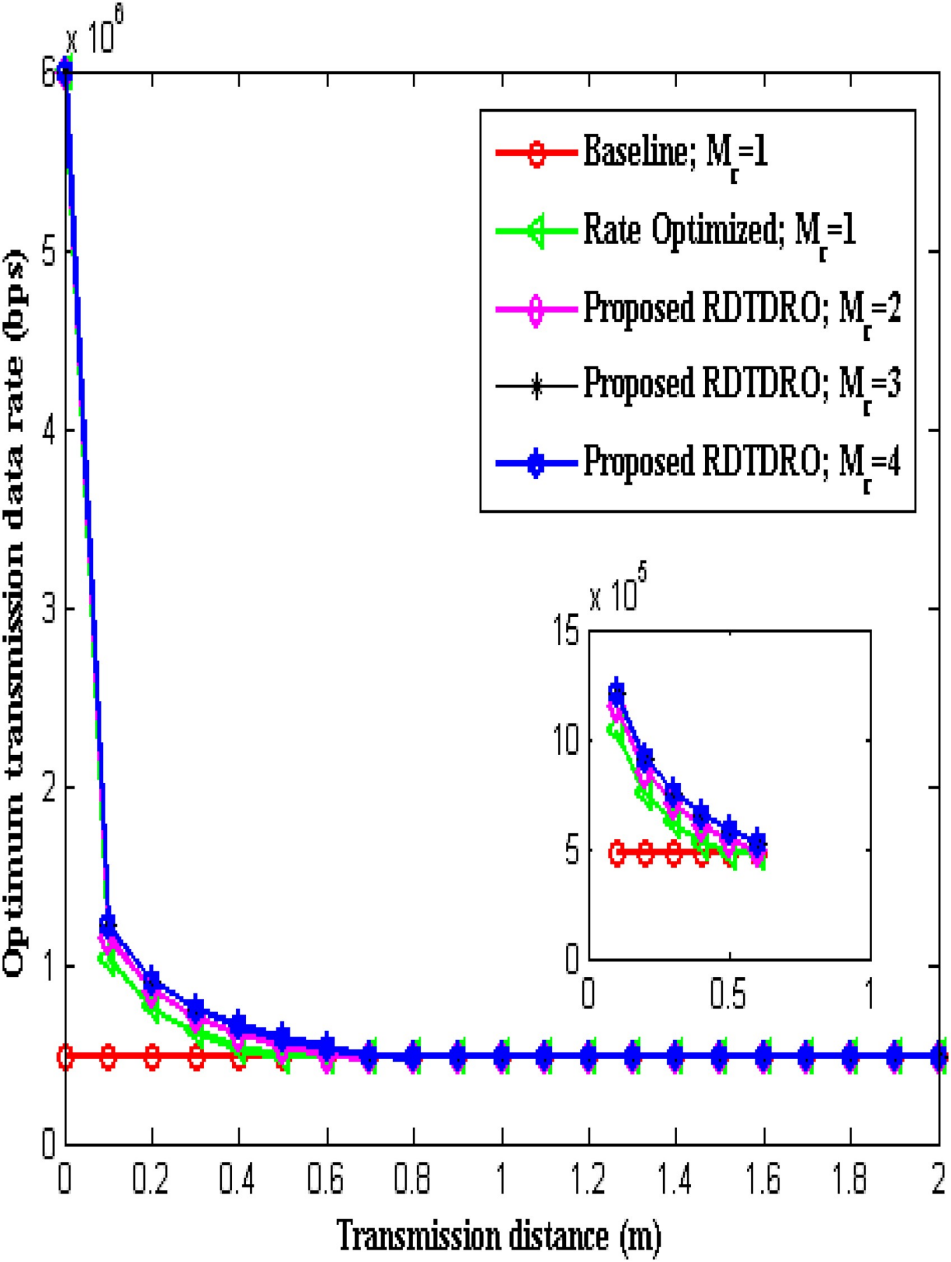
Optimum transmission data rate over transmission distance.

The optimum transmission data rate over transmission distance is illustrated in Fig 6. From the Fig, it is observed that the optimum transmission data rate decreases with increase in transmission distance for all the schemes. It is clear that the transmission data rate optimization is possible only up to *d* = 0.7 m. It is also observed that the optimum transmission data rate is higher for higher receive diversity up to *d* = 0.7 m. From *d* = 0.8 m onwards, the optimum transmission data rate becomes equal for all the schemes.

**Fig 7:**
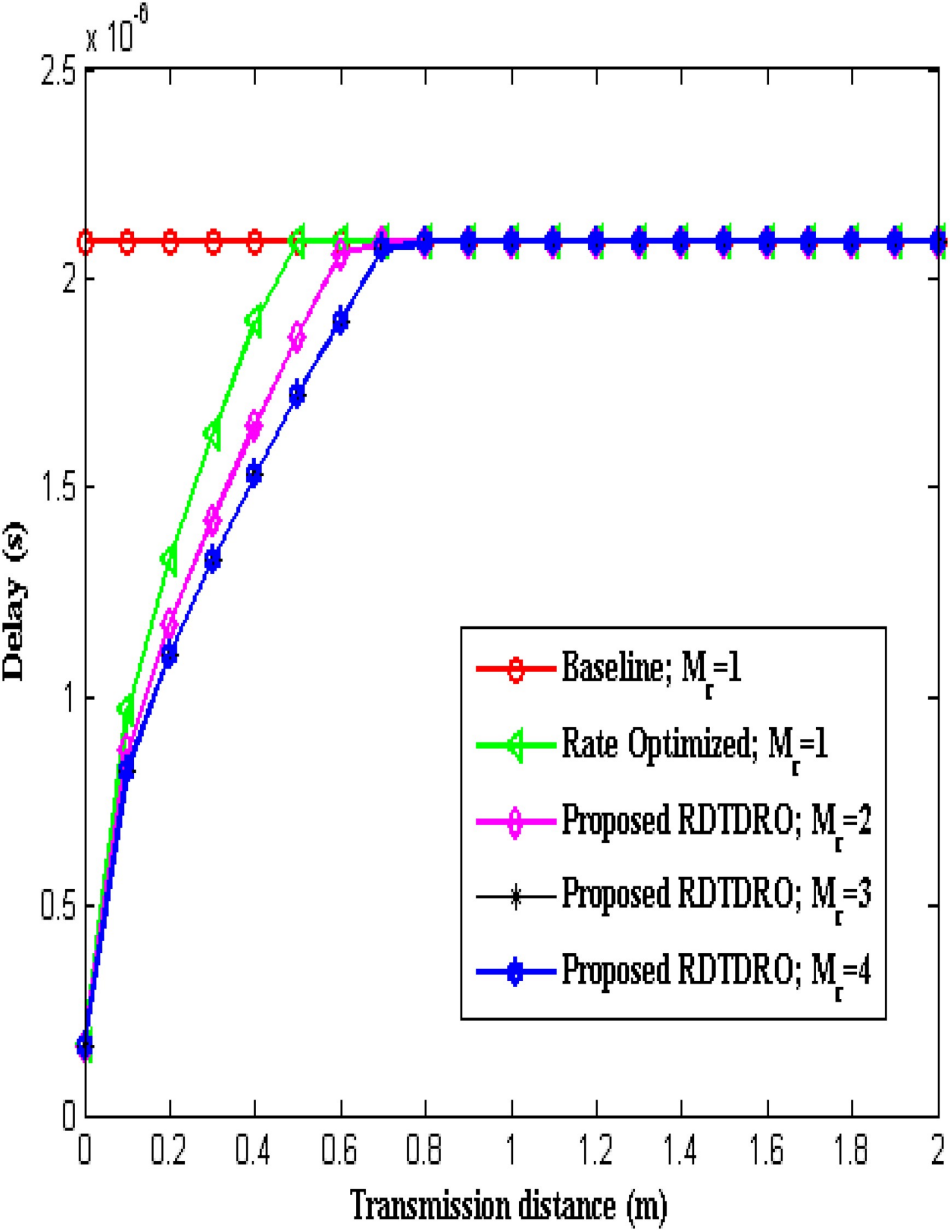
Delay over transmission distance.

Fig 7 compares the delay incurred in the transmission of *L* bits from the sensor to the BCU by employing conventional Baseline, Rate optimized schemes (with *M*_*r*_ = 1) and proposed RDTDRO schemes (with *M*_*r*_ = 2, 3 and 4) over transmission distance.

From the Fig, it is clear that the delay produced by the conventional Baseline scheme is fixed, as the transmission data rate is fixed (i.e. 480 KHz) irrespective of the transmission distance. Up to *d* = 0.4 m, the delay produced by the conventional Baseline scheme is higher when compared to other schemes, as it uses smaller transmission data rate. At *d* = 0.5 m, the delay produced by the Rate optimized scheme becomes equal to Baseline scheme, as it’s optimum transmission data rate becomes 480 KHz (as shown in Table 2). The delay produced by each one of the proposed RDTDRO schemes is lower than conventional Rate optimized scheme, as their optimum transmission data rates are higher than that of the conventional Rate optimized scheme. It is also observed that the delay produced by all the schemes are equal, above the transmission distance *d* ≥ 0.8 m, as their optimum transmission data rates become equal.

**Fig 8:**
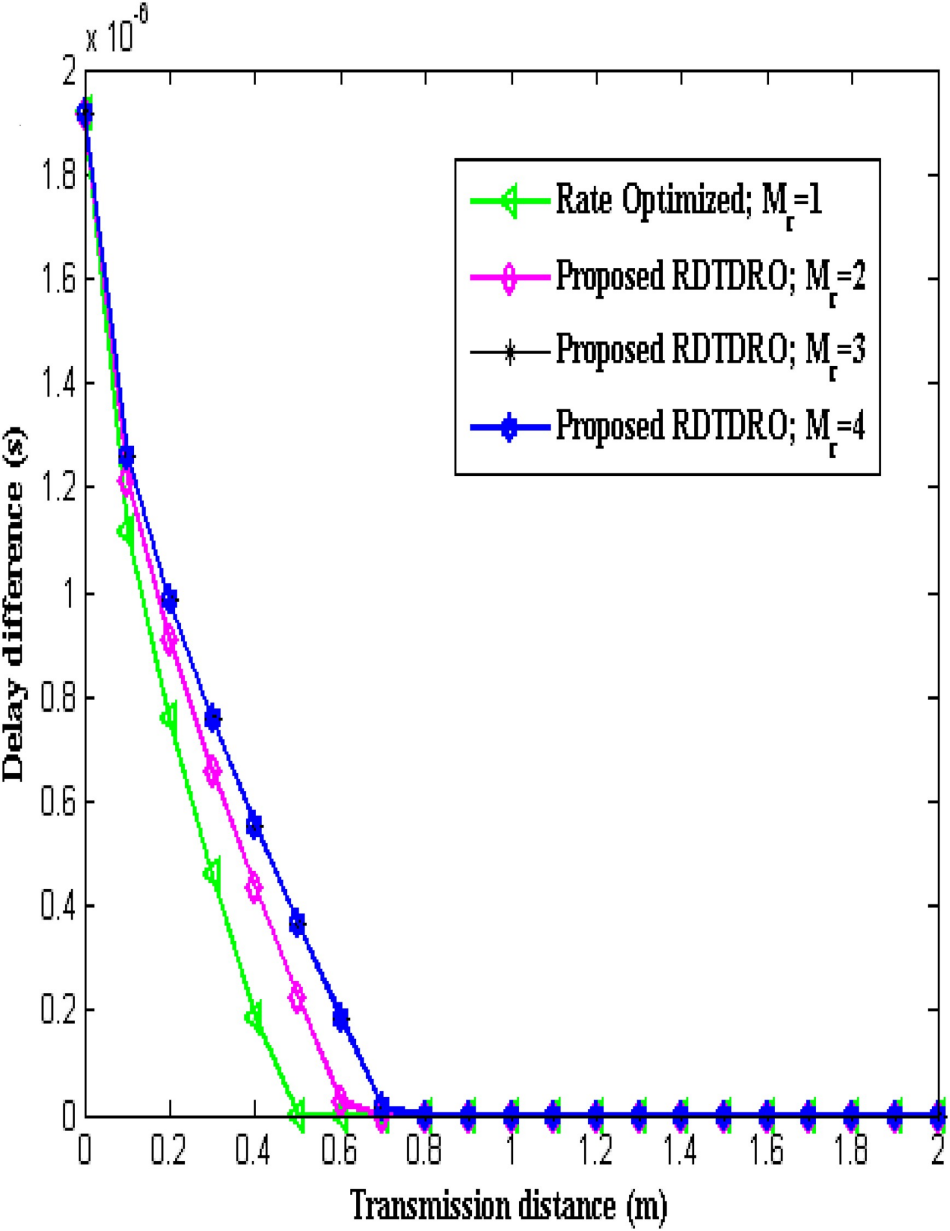
Delay difference over transmission distance.

The Fig 8 compares the difference in the delay incurred between conventional Baseline scheme and Rate optimized scheme (with *M*_*r*_ = 1) and proposed RDTDRO schemes (with *M*_*r*_ = 2, 3 and 4) in the transmission of *L* bits from the sensor to the BCU over transmission distance.

The delay of the Baseline scheme is fixed and independent of transmission distance, as the transmission data rate is fixed. The delay of the Baseline scheme is higher when compared to that of the proposed RDTDRO scheme. The proposed RDTDRO schemes utilize receive diversity in addition to transmission data rate optimization and hence their delay is lesser than Baseline scheme. The proposed work considers half-duplex operation with duty-cycling mode. The deadline time is the sum of active period and sleep period. The increase in data rate reduces the active period and reduces the transmission delay when compared to lower data rate. From the Fig, it is clear that for a given transmission distance, the delay difference increases with increase in the order of the receive diversity, as higher diversity supports higher transmission data rate. It is obvious from the Fig that the proposed RDTDRO scheme is delay efficient, as the delay difference is positive. It is also obvious that the delay difference of the conventional rate optimized scheme and proposed RDTDRO scheme decreases with increase in transmission distance. The delay difference between conventional rate optimized and Baseline scheme becomes zero from transmission distance *d* = 0.5 m onwards. The delay difference between conventional Baseline scheme and the proposed RDTDRO scheme with *M*_*r*_ = 2 becomes zero from transmission distance *d* = 0.7 m onwards. The delay difference between conventional Baseline scheme and the proposed RDTDRO scheme with *M*_*r*_ = 3 & 4 becomes zero from transmission distance *d* = 0.8 m onwards.

**Fig 9:**
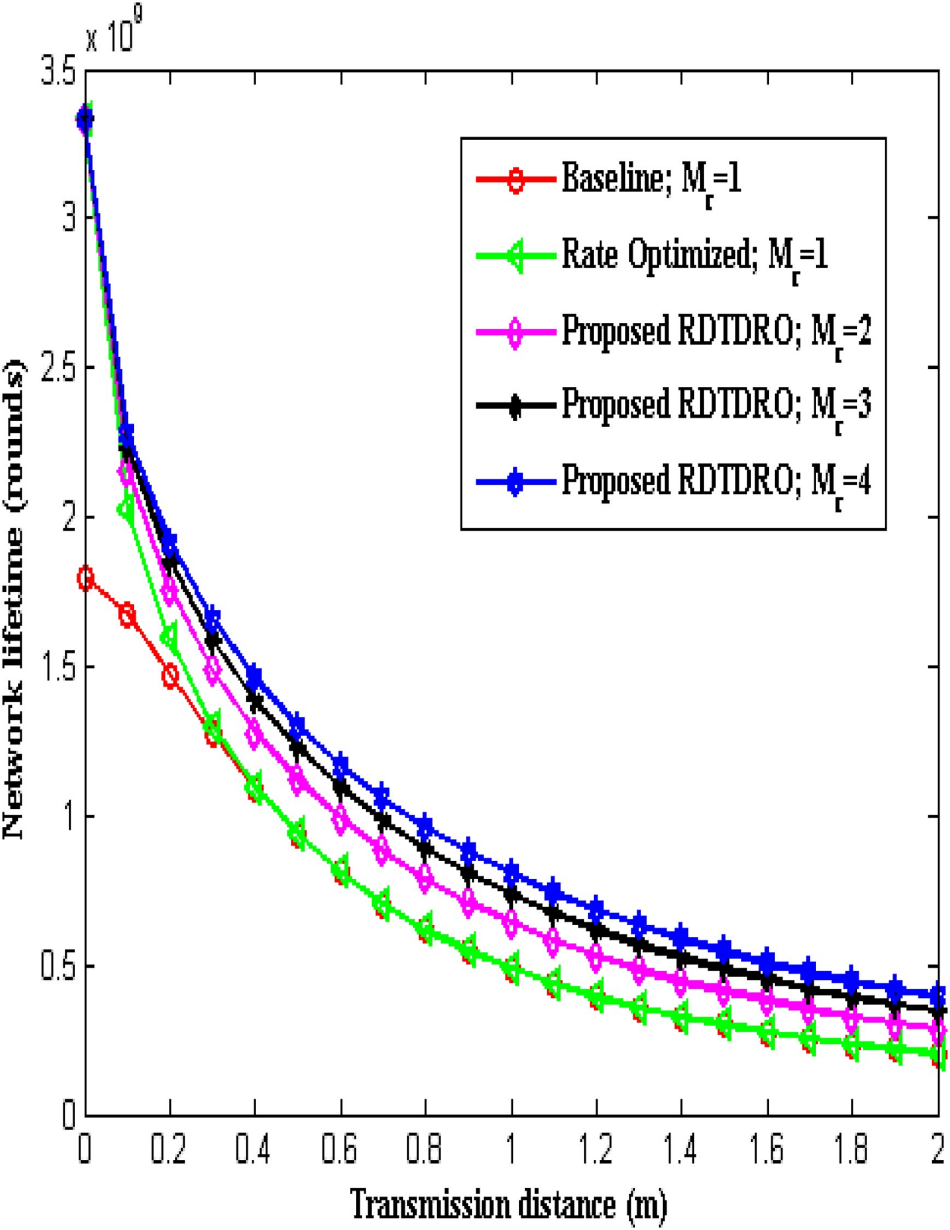
Network lifetime versus distance.

The Fig 9 compares the Network lifetime of conventional Baseline scheme and Rate optimized scheme (with *M*_*r*_ = 1) and proposed RDTDRO schemes (with *M*_*r*_ = 2, 3 and 4) in the transmission of *L* bits from the sensor to the BCU over transmission distance. The number of times a sensor node can transmit *L* = 2 kbits is considered as the network lifetime. From the Fig, it can be observed that the network lifetime of both the Baseline scheme and proposed RDTDRO scheme decreases with increase in transmission distance. From the Fig, it can also be observed that the network lifetime of the proposed RDTDRO scheme increases with increase in diversity order for a given transmission distance. The results show that at a transmission distance of *d* = 0.3 m, the proposed RDTDRO scheme with a receive diversity order of 4 achieves 1.30 times and 1.27 times improvement in network lifetime over conventional Baseline and Rate optimized schemes respectively.

## Conclusion

In this paper, Receive Diversity based Transmission Data Rate Optimization (RDTDRO) scheme is proposed to improve the network lifetime and delay efficiency of M-QAM based WBAN. The performance of proposed RDTDRO is analyzed in terms of network lifetime and delay efficiency and is compared with conventional Baseline scheme and Rate optimized scheme The results show that at a transmission distance of *d* = 0.3 m, the proposed RDTDRO scheme with a receive diversity order of 4 achieves 1.30 times and 1.27 times improvement in network lifetime over conventional Baseline and Rate optimized schemes respectively. From the results, it is also evident that at a transmission distance of 0.3 m, the proposed RDTDRO scheme with a receive diversity order of 4 is delay efficient as it achieves delay difference of 0.75 *μ*s and 0.29 *μ*s over conventional Baseline and Rate optimized schemes respectively. It can be concluded that the proposed schemes achieve significant minimization of delay and improvement in network lifetime over conventional schemes and hence proves to be efficient than conventional schemes. Therefore, the proposed scheme is suitable for applications like deep brain stimulation and electromyography. Also, the benefit of rate optimization ceases at a very shorter distance i.e. 0.8 m in the proposed scheme. However, the benefit of receive diversity exist even beyond 0.8 m also. This is evident from the simulation results. Also, higher the receive diversity, higher the benefits at higher distances. Therefore, the proposed RDTDRO scheme is suitable for higher distance as well.

## Supporting information

S1 File The optimum transmission data rates. (DOCX)

S2 File The total energy consumption. (DOCX)

S3 File Data set. (RAR)
