# Supplementary material for: Receive diversity based transmission data rate optimization for improved network lifetime and delay efficiency of Wireless Body Area Networks
Source: PLoS One. 2018 Oct 25;13(10):e0206027. doi: 10.1371/journal.pone.0206027 (PMC6201903; doi:10.1371/journal.pone.0206027)
Supplement: S1 File — (DOCX) [file pone.0206027.s001.docx]

The optimum transmission data rates for the conventional Baseline, and Rate optimized schemes and proposed RDTDRO schemes over transmission distance are shown below

| Distance  d | Optimum transmission data rate (Hz) | | | | |
| --- | --- | --- | --- | --- | --- |
|  | Baseline  Mr=1 | Rate optimized  Mr=1 | Proposed RDTDRO | | |
|  |  |  | Mr=2 | Mr=3 | Mr=4 |
| 0.0 | 480000 | 6000000 | 6000000 | 6000000 | 6000000 |
| 0.1 | 480000 | 1031900 | 1146700 | 1213400 | 1261500 |
| 0.2 | 480000 | 754800 | 852700 | 910400 | 952300 |
| 0.3 | 480000 | 615400 | 702200 | 754000 | 791800 |
| 0.4 | 480000 | 527300 | 606000 | 653300 | 688000 |
| 0.5 | 480000 | 480000 | 537600 | 581400 | 613600 |
| 0.6 | 480000 | 480000 | 485000 | 526700 | 556900 |
| 0.7 | 480000 | 480000 | 480000 | 483300 | 511700 |
| 0.8 | 480000 | 480000 | 480000 | 480000 | 480000 |
| 0.9 | 480000 | 480000 | 480000 | 480000 | 480000 |
| 1.0 | 480000 | 480000 | 480000 | 480000 | 480000 |
| 1.1 | 480000 | 480000 | 480000 | 480000 | 480000 |
| 1.2 | 480000 | 480000 | 480000 | 480000 | 480000 |
| 1.3 | 480000 | 480000 | 480000 | 480000 | 480000 |
| 1.4 | 480000 | 480000 | 480000 | 480000 | 480000 |
| 1.5 | 480000 | 480000 | 480000 | 480000 | 480000 |
| 1.6 | 480000 | 480000 | 480000 | 480000 | 480000 |
| 1.7 | 480000 | 480000 | 480000 | 480000 | 480000 |
| 1.8 | 480000 | 480000 | 480000 | 480000 | 480000 |
| 1.9 | 480000 | 480000 | 480000 | 480000 | 480000 |
| 2.0 | 480000 | 480000 | 480000 | 480000 | 480000 |
