# Supplementary material for: Receive diversity based transmission data rate optimization for improved network lifetime and delay efficiency of Wireless Body Area Networks
Source: PLoS One. 2018 Oct 25;13(10):e0206027. doi: 10.1371/journal.pone.0206027 (PMC6201903; doi:10.1371/journal.pone.0206027)
Supplement: S2 File — (DOCX) [file pone.0206027.s002.docx]

The total energy consumption corresponding to optimum transmission data rate for the conventional Baseline, and Rate optimized schemes and proposed RDTDRO schemes over transmission distance are shown below

| Distance  d | Total energy consumption (micro joules) | | | | |
| --- | --- | --- | --- | --- | --- |
|  | Baseline  Mr=1 | Rate optimized  Mr=1 | Proposed RDTDRO | | |
|  |  |  | Mr=2 | Mr=3 | Mr=4 |
| 0.0 | 0.05 | 0.027 | 0.0270 | 0.0270 | 0.0270 |
| 0.1 | 0.0538 | 0.0446 | 0.0417 | 0.0404 | 0.0396 |
| 0.2 | 0.0611 | 0.0567 | 0.0512 | 0.0487 | 0.0471 |
| 0.3 | 0.0709 | 0.0693 | 0.0606 | 0.0568 | 0.0544 |
| 0.4 | 0.0826 | 0.0824 | 0.0704 | 0.0651 | 0.0618 |
| 0.5 | 0.0961 | 0.0961 | 0.0805 | 0.0736 | 0.0695 |
| 0.6 | 0.1112 | 0.1112 | 0.0910 | 0.0825 | 0.0773 |
| 0.7 | 0.1277 | 0.1277 | 0.1020 | 0.0916 | 0.0853 |
| 0.8 | 0.1455 | 0.1455 | 0.1140 | 0.1011 | 0.0936 |
| 0.9 | 0.1647 | 0.1647 | 0.1268 | 0.1114 | 0.1024 |
| 1.0 | 0.1850 | 0.1850 | 01404 | 0.1223 | 0.1117 |
| 1.1 | 0.2065 | 0.2065 | 0.1548 | 0.1338 | 0.1215 |
| 1.2 | 0.2291 | 0.2291 | 0.1700 | 01458 | 0.1318 |
| 1.3 | 0.2528 | 0.2528 | 0.1858 | 0.1585 | 0.1426 |
| 1.4 | 0.2774 | 0.2774 | 0.2023 | 0.1717 | 0.1539 |
| 1.5 | 0.3031 | 0.3031 | 0.2195 | 0.1855 | 0.1656 |
| 1.6 | 0.3297 | 0.3297 | 0.2374 | 0.1997 | 0.1778 |
| 1.7 | 0.3573 | 0.3573 | 0.2558 | 0.2145 | 0.1904 |
| 1.8 | 0.3858 | 0.3858 | 0.2749 | 0.2297 | 0.2034 |
| 1.9 | 0.4151 | 0.4151 | 0.2945 | 0.2454 | 0.2168 |
| 2.0 | 0.4453 | 0.4453 | 0.3148 | 0.2616 | 0.2306 |
